# Improving power and accuracy of genome-wide association studies via a multi-locus mixed linear model methodology

**DOI:** 10.1038/srep19444

**Published:** 2016-01-20

**Authors:** Shi-Bo Wang, Jian-Ying Feng, Wen-Long Ren, Bo Huang, Ling Zhou, Yang-Jun Wen, Jin Zhang, Jim M. Dunwell, Shizhong Xu, Yuan-Ming Zhang

**Affiliations:** 1 State Key Laboratory of Crop Genetics and Germplasm Enhancement, Nanjing Agricultural University, Nanjing 210095, China; 2 Statistical Genomics Lab, College of Plant Science and Technology, Huazhong Agricultural University, Wuhan 430070, China; 3 School of Agriculture, Policy and Development, University of Reading, Reading RG6 6AR, United Kingdom; 4 Department of Botany and Plant Science, University of California, Riverside 20705, USA

## Abstract

Genome-wide association studies (GWAS) have been widely used in genetic dissection of complex traits. However, common methods are all based on a fixed-SNP-effect mixed linear model (MLM) and single marker analysis, such as efficient mixed model analysis (EMMA). These methods require Bonferroni correction for multiple tests, which often is too conservative when the number of markers is extremely large. To address this concern, we proposed a random-SNP-effect MLM (RMLM) and a multi-locus RMLM (MRMLM) for GWAS. The RMLM simply treats the SNP-effect as random, but it allows a modified Bonferroni correction to be used to calculate the threshold *p* value for significance tests. The MRMLM is a multi-locus model including markers selected from the RMLM method with a less stringent selection criterion. Due to the multi-locus nature, no multiple test correction is needed. Simulation studies show that the MRMLM is more powerful in QTN detection and more accurate in QTN effect estimation than the RMLM, which in turn is more powerful and accurate than the EMMA. To demonstrate the new methods, we analyzed six flowering time related traits in *Arabidopsis thaliana* and detected more genes than previous reported using the EMMA. Therefore, the MRMLM provides an alternative for multi-locus GWAS.

Genome-wide association studies (GWAS) have been widely used in genetic dissection of complex traits, especially with the development of advanced genomic sequencing technologies. The mixed linear model (MLM) method^1^,^2^ by fitting a population structure (Q) and a polygene (K), the so called Q + K model, is the most popular method used for GWAS. After the MLM of Yu *et al*.^2^ was published, many advanced MLM-based methods have been proposed^3^,^4^,^5^,^6^,^7^, primarily to improve computational efficiency. A common feature of the MLM based GWAS is the one-dimentional genome scan by testing one marker at a time. The major advantage of such a genome scanning approach is the ability to handle a large number of markers, e.g., more than a million markers. However, such a model does not facilitate good estimates of marker effects because the model is never correct if a trait is indeed controlled by multiple loci, which is the case for most complex traits. Another problem with the method is the issue of multiple test correction for the threshold value of significance test. The typical Bonferroni correction is often too conservative, so that many important loci may not pass the stringent criterion of significance test.

Most complex traits are controlled by several major loci plus numerious undetectable loci with small effects (collectively called polygenes). The one-dimentional scanning GWAS will never recover the true model due to the intrinsic limitation of the model. Multi-locus models are better alternative methods for GWAS; these include Bayesian LASSO^8^, penalized Logistic regression^9^,^10^, Elastic-Net^11^, and empirical Bayes^12^ methods. An obvious advantage of these methods is that no Bonferroni correction is required because of the multi-locus nature. Although these methods are shrinkage approaches and are supposed to be able to handle the number of markers several times larger than the sample sizes, they will fail when the number of markers is many times larger than the sample size, either due to constraint in computational time or limit in memory allocation. These models will also face the multicollinearity issue when the marker density is extremely high. Recently, Segura *et al*.^13^ has proposed a multi-locus MLM approach. However, further refinement is needed.

If the number of markers is small or moderately large and can be handled by one of the multi-locus approaches, we should consider a multi-locus method for GWAS, otherwise, a combination of the single locus genome scanning and multiple locus analysis may be considered. In the first stage, markers are scanned and selected with a low criterion of significance test. In the second stage, a multiple locus method is implemented for markers that have passed the initial screening. Statistical tests and marker effect estimation are then based on the multiple locus model. The MLM method of GWAS in the initial scanning stage treats marker effects as fixed. Goddard *et al*.^14^ proposed to treat marker effects as random, following a normal distribution with zero mean and an unknown variance. They described several advantages of the random model approach over the fixted model treatment. One advantage is that the random model approach will shrink the estimated (better called predicted) marker effects towards zero, leading to a maximum correlation between observed and predicted phenotypic values. However, Goddard *et al*.^14^ did not provide an efficient computational algorithm to estimate (or predict) marker effects.

In this study, we developed an efficient algorithm to estimate variances of the markers and predict effects of these markers. This method is called the random-SNP-effect mixed linear model (RMLM). The result of RMLM can either be treated as the final result of GWAS or used to select markers for the second stage analysis. In the second stage of GWAS, the selected markers are simultaneously evaluated in a single model using an EM empirical Bayes approach^15^. Estimation of marker effects and significance tests of these markers are performed in the second stage. This method is called the multi-locus random-SNP-effect mixed linear model (MRMLM). We demonstrate that this two-stage combined method of GWAS has significantly increased the statistical power and decreased Type 1 error compared with other methods, including the efficient mixed model analysis (EMMA).

## Results

### Statistical power for quantitative trait nucleotide (QTN) detection

**Figure 1:**
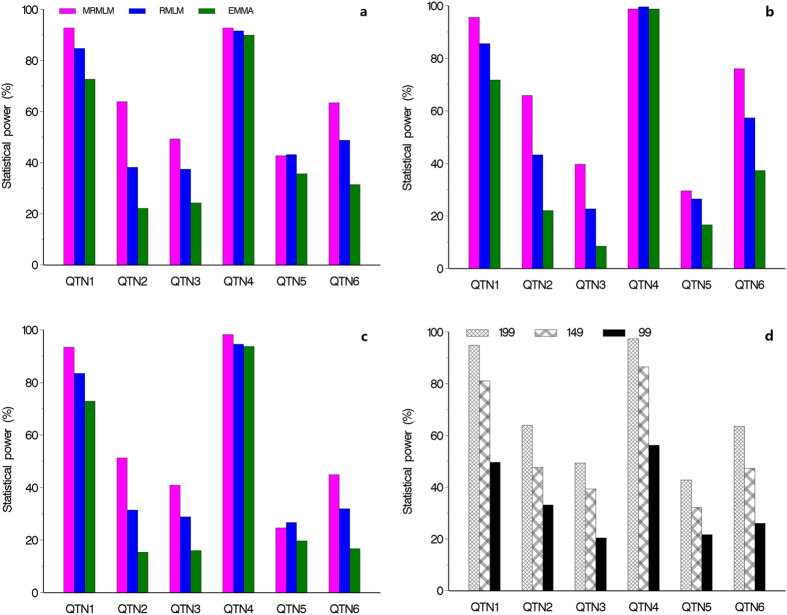
Comparison of statistical powers of six simulated QTN from three different methods of GWAS (MRMLM, RMLM and EMMA). Panel (**a**) no polgenic variance was simulated. Panel (**b**) an additive polygenic variance (explaining 0.092 of the phenotypic variance) was simulated. Panel (**c**) three epistatic QTNs each explaining 0.05 of the phenotypic variance were simulated. Panel (**d**) powers of six simulated QTNs with an additive polygenic variance obtained from the MRMLM method under three different sample sizes (199, 149 and 99).

To confirm the effectiveness of the MRMLM and RMLM methods, a series of Monte Carlo simulation experiments were carried out. Each sample was analyzed by the two new methods (MRMLM and RMLM) along the EMMA method. The significance threshold *p* value for the MRMLM method was 0.0002 (see Methods for calculation of this threshold). The corresponding threshold *p* value for the RMLM method was 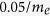 (modified Bonferroni correction for multiple tests), where 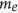 is the effective number of markers (see Methods for calculation of *m*_*e*_). The threshold *p* value for the EMMA method was 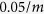 (Bonferroni correction for multiple tests), where *m* is the total number of markers. For each QTN, power was defined as the proportion of samples where the QTN was detected (*p* value smaller than the designated threshold). In the first simulation experiment where no polygenic variance was simulated, the MRMLM method has the highest power for all six QTNs simulated, followed by the RMLM method and the EMMA method (Fig. 1a and Table S1). On one occasion (QTN number 5), the RMLM method is slightly more powerful than the MRMLM method. In the second simulation experiment when an additive polygenic variance (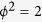 and 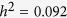) was added to the polygenic background, the same trend in power was observed where MRMLM is more powerful than RMLM and EMMA is the least powerful (Fig. 1b and Table S2). On one occasion (QTN number 4), the three methods have very similar power, with RMLM being slightly more powerful than the other two methods. In the third simulation experiement where three pairs of epistatic effects (collectively contributing 0.15 of the phenotypic variance) was added to the genetic background, again, MRMLM is the most powerful followed by RMLM and EMMA (Fig. 1c and Table S3) with an exception for QTN number 5 where RMLM is slightly more powerful than MRMLM. The sample sizes of the above three simulation experiments were all 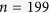. We also changed the sample size from 199 to 149 and 99 under the fourth simulation experiment with the MRMLM method. The statistical powers are demonstrated in Fig. 1(d). As expected, the statistical power has declined as we reduced the sample size (Table S4). Similar trend of power changes were also observed for different numbers of markers (Table S5).

### Accuracies of estimated QTN effects

**Figure 2:**
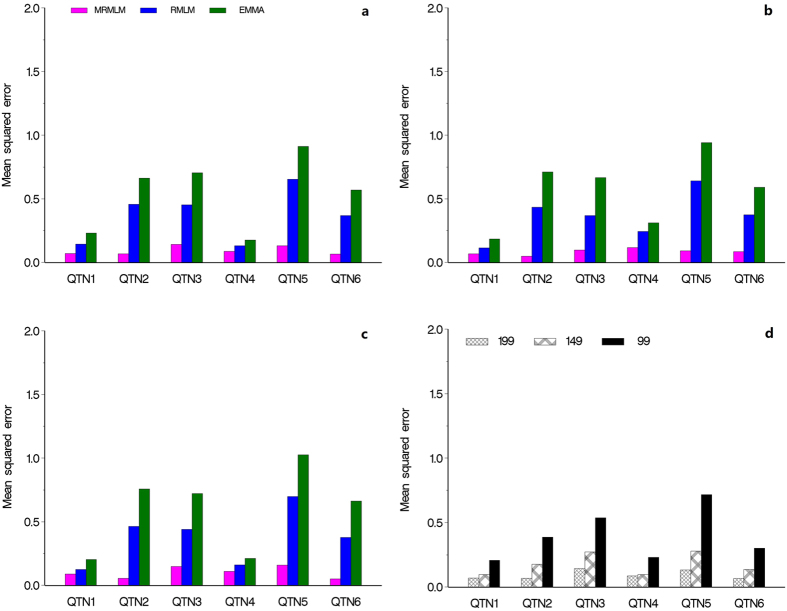
Comparison of mean squared errors of six simulated QTNs from three different methods of GWAS (MRMLM, RMLM and EMMA). Panel (**a**) no polgenic variance was simulated. Panel (**b**) an additive polygenic variance was simulated. Panel (**c**) three epistatic QTNs were simulated. Panel (**d**) mean squared errors of six simulated QTNs with an additive polygenic variance obtained from the MRMLM method under three different sample sizes (199, 149 and 99).

We used the mean squared error (MSE) to measure the accuracy of an estimated QTN effect for a particular method. We evaluated the accuracies for all the six simulated QTNs from all three methods. The MSE’s are demonstrated in Fig. 2, where panels (a), (b) and (c) represent the results from the three simulation experiments, respectively. The MRMLM method is consistently more accurate than the RMLM method, which in turn is more accurate than the EMMA method (see Tables S1–S3). Figure 2(d) shows the results of different sample sizes by the MRMLM method from the fourth simulation experiment, showing that, as expected, a large sample size is associated with a small MSE (Table S4).

### Type 1 error and ROC curve

**Figure 3:**
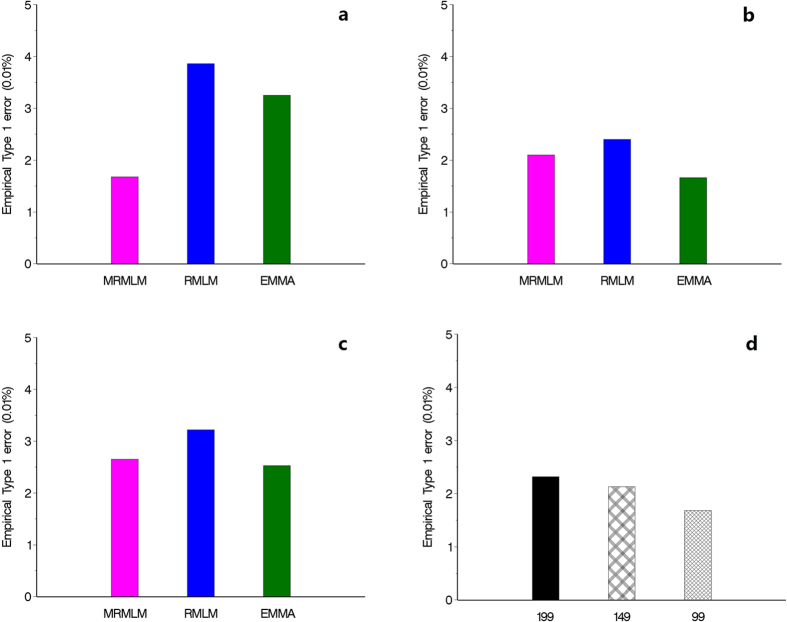
Comparison of empirical Type 1 error rates from three different methods of GWAS (MRMLM, RMLM and EMMA). Panel (**a**) no polgenic variance was simulated. Panel (**b**) an additive polygenic variance was simulated. Panel (**c**) three epistatic QTNs were simulated. Panel (**d**) empirical Type 1 error rates obtained from the MRMLM method under three different sample sizes (199, 149 and 99).

The empirical Type 1 error rates of the three methods from the three simulation experiments are illustrated in Fig. 3. Overall, the three methods have similar Type 1 errors except the first simulation experiment where EMMA has an very large Type 1 error compared with the two new methods. In the second and third simulation experiments, EMMA has the least Type 1 errors followed by the MRMLM and RMLM methods. Fig. 3(d) shows the empirical Type 1 errors of the MRMLM method from the fourth simulation experiment with three different sample sizes (199, 149 and 99), where Type 1 error has been increased with a decreased sample size.

**Figure 4:**
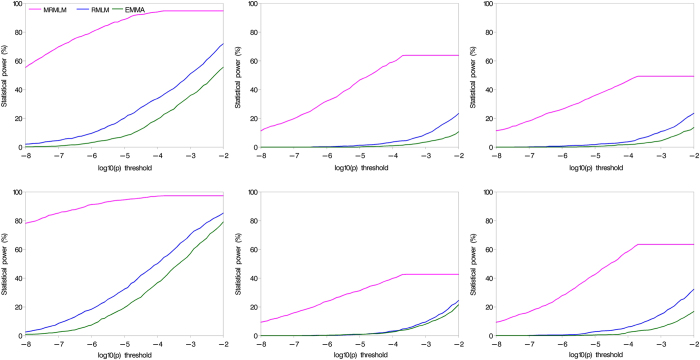
Statistical powers of six simulated QTNs from the first simulation experiement plotted against Type 1 error (in a log_10_ scale) for the three GWAS methods (MRMLM, RMLM and EMMA).

A useful way to compare different methods for their efficiencies in the detection of significant effects is the receiver operating characteristic (ROC) curve comparison. An ROC is a plot of the statistical power against the controlled Type 1 error. The higher the curve, the better the method. When sixty-one probability levels for significance, between 1E-8 to 1E-2, were inserted, the corresponding powers were calculated in the first simulation experiment. Figure 4 shows the comparison of the ROC curves from the three methods for each of the six QTNs simulated from the first simulation experiment. Clearly, the MRMLM method stands out way above the other two methods while the RMLM is better than the EMMA method when the Type 1 error is relaxed.

### Computational efficiency

When performing GWAS on the simulated data, we first scanned the genome by the single-locus RMLM method to find the association between each SNP and the trait of interest. This process took 12.78 hours (Intel Core i5 CPU 4570, 3.20 GHz, Memory 8.00G, 1000 datasets) in the first simulation experiment. The MRMLM took an additional 0.51 hours to conduct the multi-locus analysis. Although the MRMLM method requires more computing time, the high power and small MSE relative to the RMLM method are good justifications for the improved method. The EMMA method took 68.77 hours for completing the analysis for the first simulation experiment.

### Real data analyses in Arabidopsis

We analyzed six flowering time related traits of the *Arabidopsis thaliana* population published by Atwell *et al*.^16^ using all the three methods (MRMLM, RMLM and the EMMA). The numbers of SNPs significantly associated with the six traits are 29, 15, 27, 13, 22 and 14, respectively, for traits LD (days to flowering under long days), LDV (days to flowering under long days with vernalization), SD (days to flowering under short days), 0 W (days to flowering under long days with no vernalization), 2 W (days to flowering under long days for two week vernalization) and 4 W (days to flowering under long days for four week vernalization), from the MRMLM method. The corresponding numbers of associated SNPs are 8, 5, 3, 6, 6 and 7 from the RMLM method. The EMMA method only detected 1, 3, 1, 0, 1 and 2 SNPs for the above six traits (see Table S6 for details of the associated SNPs). These significantly associated SNPs for each trait were used to conduct a multiple linear regression analysis and the corresponding Bayesian information criteria (BIC) were calculated. The MRMLM method shows the lowest BIC values for all traits (Table 1), indicating that SNPs detected by the MRMLM method fit the data better than the other methods.

We found that 6, 4, 6, 2, 3 and 5 genes previously reported to be associated with the six traits are in the proximity of the SNPs detected by the MRMLM method. The corresponding numbers of genes in the vicinity of the SNPs detected by the RMLM method are 3, 3, 2, 1, 1 and 2, respectively, for the six traits. Only 2, 2, 1, 0, 0 and 1 genes are in the neighborhood of the SNPs detected by the EMMA method (see Table 2 and Table S7 for details of the genes). Clearly, the MRMLM method detected more known genes than the other two methods, indicating that this multi-locus model (MRMLM) has a higher power for QTN detection than the single-locus model (RMLM) and the EMMA method.

## Discussion

To reduce computing time required for GWAS, Zhang *et al*.^6^ proposed a P3D algorithm that fixes the polygenic-to-residual variance ratio in the genome-wide scanning step. Kang *et al*.^3^ used a matrix transformation prior to the genome-wide scanning stage and treated the scanned SNP effect as fixed. If we view the SNP effect as random, one additional variance of the QTN effect needs to be estimated, and the complexity and computing time in parameter estimation has been increased, as shown with the MLM-based approaches of Zhang *et al*.^1^ and Yu *et al*.^2^. In the present study, a new matrix transformation is constructed, the P3D algorithm is adopted, and the residual variance is estimated after the variance of the QTN effect is estimated. Therefore, only one parameter, the ratio of the QTN effect variance to the residual variance, is estimated in the genome-wide scanning stage. In doing so, the MRMLM method requires only 20% of the computing time needed by the EMMA method. More importantly, the new method performs better than EMMA in terms of high statistical power, low Type 1 error and low MSE of an estimated QTN effect.

The current GWAS method is a single-locus analysis approach under polygenic background and population structure controls. The number of tests involved is the number of markers, requiring a Bonferroni correction for multiple tests. To control the experimental error at a genome-wide level of 0.05, the significance level for each test should be adjusted as 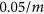, which is 5E-8 if one million markers are to be scanned. In the multi-locus model, however, there is no need for such a multiple test correction due to the multi-locus and shrinkage natures of the new method. This conclusion is also supported by the results of Monte Carlo simulation studies. We compared the result of EMMA in this study with the result reported in Atwell *et al*.^16^; fewer known genes are listed in Table 2, because some genes identified in previous studies are not significantly associated with the traits after the Bonferroni correction. If the significance level was changed to a less stringent criterion, more known genes would have been found (Table S7). We investigated the effect of the critical value on the selection of putative QTNs. Similar results were observed for the three critical values selected (0.001, 0.01 and 0.05), although the 0.01 value resulted in the marginally best performance in terms of statistical power of QTN detection and accuracy of QTN effect estimation (Table S8).

There are several multi-locus GWAS approaches already published in the literature^5^,^13^,^17^. When the number of markers is not large, all marker effects and their interactions can be included in a single model, such as the empirical Bayes method^12^. If the number of markers is large, this single model approach is not feasible. One question is how to reduce the number of parameters in a full genetic model. Zhou *et al*.^5^ developed a Bayesian sparse linear mixed model and Moser *et al*.^17^ proposed a Bayesian mixture model. Under these models, two to four common components in the mixture distribution were considered and only a few variance components were estimated. Although about 500 effects in the genetic model are finally considered after several rounds of Gibbs sampling, the computing time becomes a major concern for these Bayesian approaches. Therefore, the ideal method is to delete spurious QTN effects prior to implementing the multi-locus model. The first step of MRMLM is RMLM, which deletes the majority of the markers in advance so that only a small set of markers are left to the second stage for evaluation. The MRMLM method differs from the multi-locus method of Segura *et al*.^13^ in several aspects. First, the SNP effects are viewed as random in the MRMLM method while they are treated as fixed effects in Segura *et al*.^13^. Secondly, we adopted a simple matrix transformation technique to improve the computational efficiency while Segura *et al*.^13^ implemented an algorithm involving three complicated treatments. Finally, the MRMLM method uses one set of selected SNPs, which have *p* values less than 0.01 in the initial scanning while Segura *et al*.^13^ requires MCMC samplings.

Atwell *et al*.^16^ listed 500 most significantly associated SNPs, although some of them were not significant at the 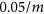 criterion. In the neighborhood of these SNPs, some genes were found to be related to the traits of interest (Table 2 and S7). In this study, 21 genes for six flowering time traits are found to be in the vicinity of the detected SNPs, consistent with results previously reported, as shown in the database ( http://www.arabidopsis.org/), the work of Atwell *et al*.^16^ and related references^18^,^19^,^20^,^21^,^22^,^23^,^24^ (Table S7). Therefore, the *Arabidopsis thaliana* GWAS results of his study appear to be reliable.

In the study of GWAS methodology, real genotypes in natural population are frequently used to conduct Monte Carlo simulation studies^1^,^2^,^6^. In this study, therefore, the real SNP dataset in Atwell *et al*.^16^ was adopted in the simulation studies. To further confirm the new methods, 200 samples with simulated genotypes derived from the minPtest R package^25^ were analyzed. As a result, similar results were found (Table S9).

## Conclusion

The RMLM simply treats the SNP effect as random, and includes new matrix transformation, fixing the polygenic-to-residual variance ratio and estimating residual variance after the variance of QTN effect is obtained. Meanwhile, it allows a modified Bonferroni correction to be used to calculate the threshold *p* value for significance tests. The MRMLM is a multi-locus model including markers selected from the RMLM, and all the effects in the model are estimated by an EM empirical Bayes method. Results from real data analyses and simulation studies show that the MRMLM has the highest power for QTN detection, the best fit for genetic model, the minimal bias in the estimation of the QTN effect, and the strongest robustness, as compared with the RMLM and the EMMA.

## Methods

### Random effect linear mixed model (RMLM)

Let **y** be a vector of phenotypic values for all individuals. The mixed linear model is


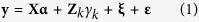


where **X** is an incident matrix for fixed (non genetic) effects, 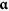 is a vector of the fixed effects, 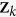 is a vector of genotype indicators for the *k*th SNP that are coded as 1, 0 and −1, for one of the two homozygotes, the heterozygote and the other homozygote, respectively, 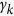 is the effect of marker *k* with an assumed normal distribution of mean zero and variance 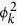, 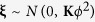 is a vector of polygenic effects with a multivariate normal distribution of mean zero and variance 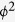 described by a covariance structure **K**, **ε** is a vector of residual errors with a 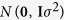 distribution and 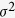 is the residual variance. The expectation of **y** is 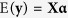 and the variance is


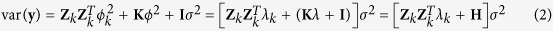


where 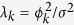 and 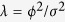 are variance ratios, and 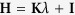.Various methods of inferring a kinship matrix have been proposed. In this study we used a marker-inferred kinship matrix^26^ defined as


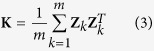


In the single-locus RMLM, the polygenic variance ratio *λ* is only estimated once under a pure polygenic model (the null model) prior to the marker scanning stage. The estimated variance ratio, 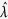, is then treated as a constant when markers are scanned. This approach has been called GWAS with population parameters previously defined (P3D)^6^. The original P3D was implemented when 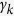 was treated as a fixed effect. In this study, 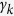 is treated as a random effect, which presents a great challenge in computation. However, we adopted a new algorithm to ease the computation, as described in the following paragraph.

Let us perform eigen decomposition for **K** so that 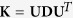, where 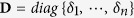 is a diagonal matrix for the eigenvalues and **U** is an *n *× *n* matrix for the eigenvectors. Let 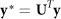, 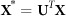 and 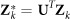 be transformed variables so that


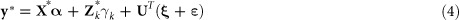


The expectation of 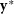 is 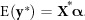. The variance-covariance matrix of 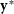 is


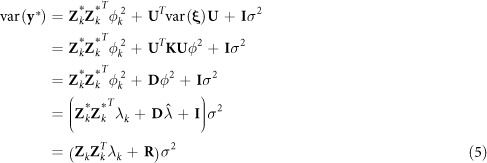


where 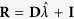 is a known diagonal matrix. Let 
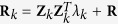
 be the general covariance structure. After absorbing 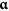 and *σ*^2^, we have the following profiled restricted log likelihood function,


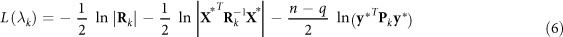


where *q* is the rank of matrix **X** and


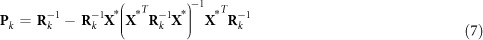


This likelihood function contains only one unknown parameter *λ*_*k*_. The Newton algorithm for *λ*_*k*_ is


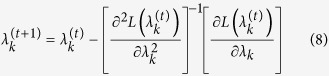


Once the iteration process converges, the solution is a single-locus RMLM estimate of *λ*_*k*_, denoted by 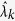. Note that the likelihood function involves 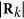 and 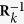, which are very expensive to compute. However, the special structure of 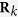 allows us to implement the Woodbury matrix identities^27^ for calculating 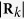 and 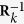. As a result, the random model approach does not present a substantial increase in computational time.

Given 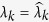, the estimates of 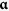 and *σ*^2^ are


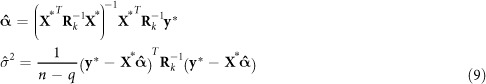


The best linear unbiased prediction (BLUP) of γ_*k*_ is also the conditional expectation of γ_*k*_ given 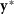 and has the following expression,


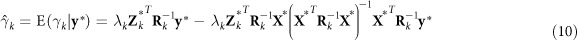


The conditional variance is


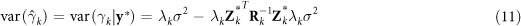


Under the single-locus RMLM approach, we first estimate 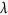 and then fix it at 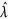 to estimate *λ*_*k*_ and scan all markers by testing *λ*_*k*_ = 0 for each SNP. The null hypothesis test for 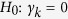 or 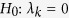 in the single-locus RMLM is implemented using a Wald test,


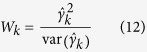


The p-value of this Wald test is calculated using


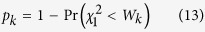


where 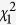 is a Chi-square variable with one degree of freedom.

Because the estimated marker effects under the random model are shrunken towards zero, we are able to use a modified Bonferroni correction to find the threshold *p* value for genome-wide significance tests^28^. This modified Bonferroni correction uses an effective number of markers to adjust for multiple tests so that the threshold *p* value is 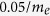, where


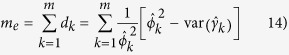


is the effective number of markers.

### Multi-locus random effect mixed linear model (MRMLM)

The single marker RMLM method described above can also be considered as an intial screening step for a new multi-locus random effect mixed linear model (MRMLM) that is described here. We use a less stringent criterion for the initial stage screening from RMLM for all markers that have *p* values smaller than 0.01. In addition, consecutive markers passing the 0.01 threshold around an already selected marker (±20 kb for real data analysis and ± 1 kb for simulated data analysis) are eliminated to reduce collinearity among selected markers. Only these selected markers are included in the muti-locus model for further evaluation, including estimation of marker effects and significane tests. Due to the shrinkage nature, the majority of markers will be eliminated in the intitial screening. Therefore, the number of markers left in the second stage analysis is often a small subset of all markers, say a few hundred or a few thousand at most. Among the remaining markers, all those that passed the modified Bonferroni correction are used to conduct a likelihood ratio test (LRT), and the others are treated as random. If the LOD score for one marker in the LRT is more than 1.50, this marker is treated as fixed, or it is viewed as random. This small number of surviving markers are then included in a single multi-locus model. We propose to use the EM empirical Bayes (EMEB) method^15^ because this method also provides a significance test for each marker (likelihood ratio test), while the LASSO method does not have a default method to perform such a test. The EMEB method is also a random model approach because each random marker effect is assigned an empirical distribution with a variance. Because the model is multi-locus in nature, there is no requirement for Bonferroni correction. Therefore, the original 0.05 threshold may be used for significance test. Considering that all markers are selected in the first stage, we decided to place a slightly more stringent criterion of 0.0002, which is converted from LOD score 3.0 of the test statistics using 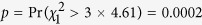, where 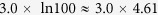 is converted from LOD 3 to its corresponding likelihood ratio test, which, under the null hypothesis, follows a Chi-square distribution with one degree of freedom.

### Efficient mixed model analysis (EMMA)

This is an existing method for GWAS^3^ and used as a gold standard for comparison. This method is the fixed model version of the original MLM, in which 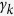 was treated as a fixed effect with no distribution assigned. The method was implemented in the R software package EMMA ( http://mouse.cs.ucla.edu/emma/). The threshold of *p* value was set as 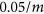 after Bonferroni correction for multiple tests, where *m* is the number of markers.

### Simulation experiments

In the first four simulation experiments, all the SNP genotypes were derived from the 216130 SNPs in Atwell *et al*.^16^. All the SNPs between 11226256 and 12038776 bp on Chr. 1, between 5045828 and 6412875 bp on Chr. 2, between 1916588 and 3196442 bp on Chr. 3, between 2232796 and 3143893 bp on Chr. 4, and between 19999868 and 21039406 bp on Chr. 5 were used to conduct simulation studies. The sample size was the number of individuals in Atwell *et al*.^16^, namely 199. In the first simulation experiment, six QTNs were simulated and placed on the SNPs with allelic frequencies of 0.30; their heritabilities were set as 0.10, 0.05, 0.05, 0.15, 0.05 and 0.05, respectively; and their positions and effects are listed on Table S1. The average was set at 10.0; and residual variance was set at 10.0. Empirical statistical power for each QTN was calculated as the proportion of samples in which the *p* value is smaller than the designated threshold *p* value. A QTN detected within 1 kb of the simulated QTN was considered a true QTN. Empirical Type 1 error for each method was defined as the proportion of significant markers (excluding the markers overlapping with the six QTNs) over all markers with zero effects. In addition to power and Type 1 error, we also evaluated the mean square error (MSE) for each of the six simulated QTNs. For the *i*th QTN for 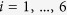, the MSE is defined as


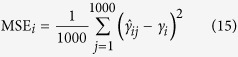


where 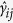 is the estimated effect of QTN *i* from the *j*th sample and 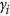 in the true effect of QTN *i*. A method with a small MSE is generally more preferable than a method with a large MSE.

To investigate the effect of the polygenic (small effect genes) background on the MRMLM and RMLM methods, the polygenic effect was simulated by multivariate normal distribution 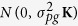, where 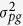 is the polygenic variance, and 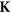 is the kinship coefficient matrix between a pair of individuals. Here 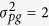, so 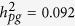. The QTN size (*h*^2^), average, residual variance, and other values were the same as those in the first simulation experiment.

To investigate the effect of epistatic background on the MRMLM and RMLM methods, three epistatic QTNs each with 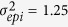 and 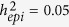 were simulated. The first one was placed between 3063784 bp on Chr. 4 and 5227063 bp on Chr. 2; the second one was placed between 5986135 bp on Chr. 2 and 2031781 bp on Chr. 3; and the third one was placed between 2668059 bp on Chr. 3 and 11824678 bp on Chr. 1. The QTN sizes (*h*^2^), average, residual variance, and other values were also the same as those in the first simulation experiment.

### The *Arabidopsis thaliana* data

We also analyzed the well-known *Arabidopsis thaliana* data published by Atwell *et al*.^16^. The data contain 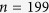 accessions with 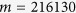 genotyped SNPs. Six flowering time related quantitative traits were analyzed using all the three methods (MRMLM, RMLM and EMMA). The six traits are: LD, LDV, SD, 0 W, 2 W and 4 W. These data were downloaded from the following website: http://www.arabidopsis.usc.edu/. We developed our own software to implement the data analysis (see Software S1).

## Additional Information

**How to cite this article**: Wang, S.-B. *et al*. Improving power and accuracy of genome-wide association studies via a multi-locus mixed linear model methodology. *Sci. Rep.* **6**, 19444; doi: 10.1038/srep19444 (2016).

## Supplementary Material

Supplementary Information

## Figures and Tables

**Table 1:** Goodness of fit (log likelihood and BIC) for SNPs detected by three methods (MRMLM, RMLM and EMMA), where a lower value indicates a better fit.

Trait^a^	−Log likelihood value			Bayesian information criterion (BIC)		
	MRMLM	RMLM	EMMA	MRMLM	RMLM	EMMA
LD	−215.9	153.8	284.6	−67.5	194.8	289.7
SDV	−337.2	−150.1	−119.9	−260.4	−124.5	−104.5
SD	−367.9	55.3	113.1	−230.5	70.5	118.2
0W	−42.4	94.5	226.2	21.5	124.0	226.2
2W	−140.6	104.4	216.4	−30.1	134.6	221.4
4W	−122.4	24.5	105.3	−55.5	57.9	114.9

^a^LD: days to flowering under long days; LDV: days to flowering under long days with vernalization; SD: days to flowering under short days; 0W: days to flowering under long days for no vernalization; 2W: days to flowering under long days for 2 weeks vernalization; 4W: days to flowering under long days for 4 weeks vernalization.

**Table 2:** Genes detected for six flowering time traits in *Arabidopsis thaliana* using three methods (MRMLM, RMLM and EMMA).

Trait	Gene detected	Chr	SNP (bp)	MRMLM			RMLM			EMMA			Reference
				LOD	Effect	r^2^(%)	P-value	Effect	r^2^(%)	P-value	Effect	r^2^(%)	
LD	*AT1G23000*	1	8128350	4.12	−0.055	0.56							
	*SVP*	2	9588685				4.47E-10	−0.397	23.36	2.78E-9	−0.407	24.59	18
	*AGL17*	2	9611587	8.83	−0.104	1.67	7.11E-7	−0.262	10.56				19
	*ETC3*	4	454542	4.70	−0.066	0.72							20
	*GA1*	4	1260796	3.06	−0.059	0.63							21
	*AT4G14385*	4	8291057	6.18	−0.060	0.66							
	*FLC*	5	3188328	9.48	−0.097	1.35	5.08E-7	−0.248	8.73	8.82E-7	−0.258	9.45	22
LDV	*CENH3*	1	164375	6.30	−0.049	3.37							
	*SVP*	2	9588685	10.30	−0.049	2.98	1.61E-6	−0.111	15.43				18
	*CKB4*	2	18446546	5.75	−0.053	2.05	2.66E-8	−0.152	16.80	5.74E-8	−0.158	18.15	23
	*DOG1*	5	18599929	7.25	−0.058	4.77	2.04E-7	−0.083	9.82	2.71E-7	−0.087	10.87	
SD	*AT1G52930*	1	19713470	6.21	−0.049	1.27							
	*HEN2*	2	2916675	3.83	0.030	0.53							
	*EDA8*	4	153402	6.47	−0.042	1.13	2.46E-8	−0.132	11.26	6.74E-8	−0.136	12.00	
	*ETC3*	4	458226	10.70	−0.070	2.03	1.20E-6	−0.132	7.18				20
	*IDL3*	5	3051259	6.23	−0.037	0.87							
	*AT5G19430*	5	6546055	4.66	−0.068	1.36							
0W	*AGL18*	3	21239134	6.24	−0.111	3.13							24
	*DOG1*	5	18592535				1.83E-6	−0.274	12.72				
	*DOG1*	5	18595015	11.87	−0.145	3.56							
2W	*ANP1*	1	2899659	8.11	−0.081	1.55							
	*ETC3*	4	454542	7.18	−0.089	1.72	4.30E-7	−0.207	9.21				20
	*DCL4*	5	6846957	3.36	−0.082	0.99							
4W	*ATPERK12*	1	8341601	4.65	−0.070	1.34							
	*SVP*	2	9588685				3.55E-9	−0.354	28.25	1.97E-8	−0.365	30.03	18
	*AT2G30600*	2	13031229	9.65	−0.107	3.00							
	*C3HC4*	5	6546259	5.94	−0.132	2.75							
	*AT5G45190*	5	18264316	7.06	−0.071	1.33							
	*DOG1*	5	18607728	10.09	−0.164	4.69	8.13E-7	−0.263	12.09				

r^2^: Proportion of phenotypic variance contributed by the gene.
