# Supplementary material for: Improving power and accuracy of genome-wide association studies via a multi-locus mixed linear model methodology
Source: Sci Rep. 2016 Jan 20;6:19444. doi: 10.1038/srep19444 (PMC4726296; doi:10.1038/srep19444)
Supplement: Supplementary Information [file srep19444-s1.pdf]

Improving power and accuracy of genome-wide association studies via a multi-locus mixed linear model methodology

Shi-Bo Wang[1,2], Jian-Ying Feng[1], Wen-Long Ren[1], Bo Huang[1], Ling Zhou[1], Yang-Jun Wen[1],
Jin Zhang[1], Jim M. Dunwell[3], Shizhong Xu[4] & Yuan-Ming Zhang[1,2]

[1]State Key Laboratory of Crop Genetics and Germplasm Enhancement, Nanjing Agricultural University, Nanjing 210095, China, [2]Statistical Genomics Lab, College of Plant Science and Technology, Huazhong Agricultural University, Wuhan 430070, China, [3]School of Agriculture, Policy and Development, University of Reading, Reading RG6 6AR, United Kingdom, [4]Department of Botany and Plant Science, University of California, Riverside 20705, USA.

Table S1 | Comparison of powers and mean squared errors (MSE) for three GWAS methods (MRMLM, RMLM and EMMA) from the first simulation experiment where six QTNs were simulated.

QTN	True value				MRMLM			RMLM			EMMA		
	Chr.	Position (bp)	Effect	r^2 (%)	Power (%)	Effect	MSE	Power (%)	Effect	MSE	Power (%)	Effect	MSE
1	1	11298364	1.4762	10	94.8	1.5412	0.0701	84.7	1.7694	0.1445	72.7	1.9084	0.2309
2	1	11655607	1.0438	5	63.9	1.2083	0.0674	38.2	1.6772	0.4582	22.1	1.8464	0.6634
3	2	5066968	1.0438	5	49.3	1.3291	0.1423	37.5	1.6921	0.4538	24.2	1.8675	0.7049
4	2	5134228	1.8080	15	97.3	1.7922	0.0867	91.6	2.0617	0.1308	89.9	2.1590	0.1766
5	2	5464675	1.0438	5	42.7	1.3178	0.1318	43.2	1.8328	0.6532	35.7	1.9862	0.9128
6	2	6137189	1.0438	5	63.5	1.2072	0.0659	48.8	1.6263	0.3688	31.4	1.7836	0.5704

Table S2 | Comparison of powers and mean squared errors (MSE) for three GWAS methods (MRMLM, RMLM and EMMA) from the second simulation experiment where six QTNs plus a polygene were simulated.

QTN	True value				MRMLM			RMLM			EMMA		
	Chr.	Position (bp)	Effect	r^2 (%)	Power (%)	Effect	MSE	Power (%)	Effect	MSE	Power (%)	Effect	MSE
1	1	11298364	1.6171	10	95.6	1.5845	0.0692	85.6	1.8629	0.1143	71.7	1.9993	0.1852
2	1	11655607	1.1435	5	65.9	1.2283	0.0484	43.3	1.7809	0.4344	22.1	1.9752	0.7121
3	2	5066968	1.1435	5	39.7	1.3408	0.0973	22.7	1.7331	0.3695	8.5	1.9486	0.6665
4	2	5134228	1.9806	15	98.8	2.0872	0.1177	99.6	2.3817	0.2435	98.8	2.4713	0.3111
5	2	5464675	1.1435	5	29.6	1.3282	0.0924	26.5	1.9281	0.6405	16.6	2.1039	0.9415
6	2	6137189	1.1435	5	76.1	1.3339	0.0861	57.3	1.7288	0.3743	37.3	1.8969	0.5915

Table S3 | Comparison of powers and mean squared errors (MSE) for three GWAS methods (MRMLM, RMLM and EMMA) from the third simulation experiment where six QTNs, a polygene and three pairs of epistatic effects were simulated.

QTN	True value				MRMLM			RMLM			EMMA		
	Chr.	Position (bp)	Effect	r^2 (%)	Power (%)	Effect	MSE	Power (%)	Effect	MSE	Power (%)	Effect	MSE
1	1	11298364	1.7310	10	93.4	1.7479	0.0886	83.5	1.9855	0.1247	72.9	2.1286	0.2044
2	1	11655607	1.2240	5	51.3	1.3319	0.0550	31.4	1.8845	0.4628	15.4	2.0837	0.7576
3	2	5066968	1.2240	5	40.9	1.5137	0.1497	28.8	1.8711	0.4407	16.0	2.0656	0.7218
4	2	5134228	2.1201	15	98.3	2.1022	0.1114	94.6	2.3934	0.1605	93.8	2.5027	0.2131
5	2	5464675	1.2240	5	24.6	1.5209	0.1590	26.7	2.0423	0.6987	19.7	2.2242	1.0254
6	2	6137189	1.2240	5	44.9	1.2886	0.0507	31.9	1.8111	0.3771	16.7	2.0238	0.6615

Table S4 | Statistical powers and mean squared errors (MSE) of the MRMLM method under three different sample sizes (99, 149 and 199).

QTN	True value				99			149			199		
	Chr.	Position (bp)	Effect	r^2 (%)	Power (%)	Effect	MSE	Power (%)	Effect	MSE	Power (%)	Effect	MSE
1	1	11298364	1.4762	10	49.6	1.7652	0.2066	81.0	1.5990	0.0963	94.8	1.5412	0.0701
2	1	11655607	1.0438	5	33.1	1.5850	0.3874	47.6	1.3781	0.1752	63.9	1.2083	0.0674
3	2	5066968	1.0438	5	20.4	1.6996	0.5371	39.3	1.4720	0.2713	49.3	1.3291	0.1423
4	2	5134228	1.8080	15	56.2	2.0754	0.2307	86.5	1.8133	0.0965	97.3	1.7922	0.0867
5	2	5464675	1.0438	5	21.6	1.8139	0.7172	32.1	1.4828	0.2781	42.7	1.3178	0.1318
6	2	6137189	1.0438	5	26.0	1.5206	0.3011	47.2	1.3322	0.1372	63.5	1.2072	0.0659

Table S5 | Powers and mean squared errors (MSE) of the MRMLM method under two different numbers of markers (10,000 and 216,130).

QTN	True value				10,000			216,130		
	Chr.	Position (bp)	Effect	r^2 (%)	Power (%)	Effect	MSE	Power (%)	Effect	MSE
1	1	11298364	1.4762	10	94.8	1.5412	0.0701	92.0	1.3520	0.2941
2	1	11655607	1.0438	5	63.9	1.2083	0.0674	48.0	1.1234	0.0927
3	2	5066968	1.0438	5	49.3	1.3291	0.1423	39.0	1.2079	0.1216
4	2	5134228	1.8080	15	97.3	1.7922	0.0867	92.0	1.4864	0.0862
5	2	5464675	1.0438	5	42.7	1.3178	0.1318	59.0	1.1660	0.1019
6	2	6137189	1.0438	5	63.5	1.2072	0.0659	42.0	0.9997	0.0510

Table S6 | Detected SNPs significantly associated with the six flowering time traits in *Arabidopsis thaliana* using three methods (MRMLM, RMLM and EMMA).

Trait	Chr	SNP (bp)	MRMLM		RMLM		EMMA	
			LOD score	Effect	P-value	Effect	P-value	Effect
LD	1	2779077	9.07	-0.099				
	1	8128350	4.12	-0.055				
	1	11738770	4.61	0.056				
	1	16590690	4.83	0.072				
	1	19306376	8.93	-0.092				
	2	3908570	8.38	-0.084				
	2	9588685			4.47E-10	-0.397	2.78E-9	-0.407
	2	9611587	8.83	-0.104	7.11E-7	-0.262		
	2	13168846			1.82E-6	-0.271		
	3	7548675	4.97	0.055				
	3	10857602	5.60	0.070				
	3	15980134	3.53	-0.048				
	4	109403	5.37	-0.071				
	4	429928	5.56	-0.074	3.43E-7	-0.230		
	4	454542	4.70	-0.066				
	4	1260796	3.06	-0.059				

	4	2579595	5.29	-0.078		
	4	6265724	4.43	-0.055		
	4	6904769	6.65	-0.071		
	4	8291057	6.18	-0.060		
	4	8949791	3.74	0.067		
	4	15531205	7.00	0.072		
	4	15645842	17.92	-0.170	1.32E-6	-0.283
	4	17324466	3.99	0.057		
	5	2047037	9.12	0.118	1.28E-6	0.282
	5	3188328	9.48	-0.097	5.08E-7	-0.248
	5	12782295	3.19	-0.052		
	5	19469740	6.12	-0.080		
	5	21844046	8.82	-0.072		
	5	23928370	9.98	-0.096		
	5	25407568	5.20	-0.075	7.83E-7	-0.268
LDV	1	164375	6.30	-0.04929		
	1	10114810	3.25	0.026		
	1	26552074	10.09	0.046		
	2	5851520	5.61	0.034		

	2	9588685	10.30	-0.049	1.61E-6	-0.111		
	2	18446546	5.75	-0.053	2.66E-8	-0.152	5.74E-8	-0.158
	3	16092326	7.82	-0.072				
	3	19639423	5.12	0.034				
	3	21717659	4.33	-0.023				
	4	424505	3.78	-0.031				
	4	10528698	5.83	-0.038	1.27E-6	-0.069		
	4	16040939	3.48	-0.047				
	5	7704503	3.28	-0.026				
	5	18599929	7.25	-0.058	2.04E-7	-0.083	2.71E-7	-0.087
	5	25290046	5.71	-0.060	1.12E-7	-0.108	2.35E-9	-0.126
SD	1	7450037	7.59	-0.043				
	1	10298977	5.69	-0.061				
	1	19483362	6.01	0.042				
	1	19713470	6.21	-0.049				
	1	23894668	6.51	0.058				
	2	1977590	5.43	-0.055				
	2	2916675	3.83	0.030				
	2	3037271	3.75	-0.032				

2	13184349	7.30	-0.056				
2	18992837	6.35	-0.048				
3	10516222	9.30	0.045				
3	18021311	4.04	0.032				
4	153402	6.47	-0.042	2.46E-8	-0.132	6.74E-8	-0.136
4	458226	10.70	-0.070	1.20E-6	-0.132		
4	5969868	4.90	0.029				
4	6971183	9.59	-0.059				
4	14263080	4.72	0.031				
4	15645842	17.70	-0.089	8.60E-7	-0.152		
5	441370	3.35	-0.036				
5	3051259	6.23	-0.037				
5	3408398	4.27	-0.035				
5	6546055	4.66	-0.068				
5	7003093	8.12	0.053				
5	10031343	4.62	0.040				
5	17218821	3.47	-0.029				
5	18363740	6.30	0.035				
5	21499321	7.06	-0.042				

W0	2	10254298	7.31	-0.107		
	2	12724525	6.71	0.114		
	3	10850207	7.01	-0.136		
	3	21239134	6.24	-0.111		
	4	429928	3.54	-0.081	1.18E-7	-0.213
	4	1420057	7.83	-0.160	1.35E-6	-0.234
	4	2240573	4.25	0.093		
	4	5188778	3.94	-0.083		
	4	8012841	3.21	0.090		
	5	4867634	6.06	-0.119	4.30E-7	-0.266
	5	5759583	3.37	0.116		
	5	18592535			1.83E-6	-0.274
	5	18595015	11.87	-0.145	1.83E-6	-0.274
	5	18598041			1.83E-6	-0.274
	5	20745711	3.29	-0.083		
W2	1	2899659	8.11	-0.081		
	1	3978063	13.38	-0.155	2.11E-7	-0.315
	1	10534477	3.70	0.093		
	1	11014292	3.59	-0.065		

1	14112717	3.42	0.060				
2	5890727	3.68	0.049				
2	9762418	5.20	0.083				
2	13912889	6.98	-0.110	9.18E-9	-0.328	4.91E-8	-0.338
3	5579959	13.92	-0.185	1.19E-6	-0.283		
3	11837536	3.02	0.064				
3	15772202	4.03	0.061				
3	23090917	6.12	-0.124	6.56E-7	-0.282		
4	454422			1.08E-6	-0.199		
4	454542	7.18	-0.089	4.30E-7	-0.207		
4	15645842	4.17	-0.104				
5	6289819	6.39	-0.086				
5	6846957	3.36	-0.082				
5	12775299	4.33	-0.055				
5	16849870	3.05	-0.053				
5	17426385	4.28	-0.080				
5	18614346	3.38	-0.057				
5	22900714	6.74	0.070				
5	25211216	3.26	0.052				

W4	1	3978063	15.82	-0.207	5.52E-8	-0.341	1.61E-7	-0.355
	1	8341601	4.65	-0.070				
	2	9588685			3.55E-9	-0.354	1.97E-8	-0.365
	2	13031229	9.65	-0.107				
	2	13853405			1.49E-6	-0.276		
	2	14971763			2.46E-7	-0.392		
	3	1571179	4.63	0.076				
	3	5579959	14.64	-0.173	5.82E-7	-0.294		
	3	10855475	4.27	0.088	2.64E-7	0.174		
	3	14573793	4.62	0.071				
	3	16896336	3.99	0.081				
	4	15358265	8.10	-0.102				
	5	6546259	5.94	-0.132				
	5	18264316	7.06	-0.071				
	5	18607728	10.09	-0.164	8.13E-7	-0.263		
	5	19525511	3.08	-0.084				
	5	21145900	6.85	-0.092				

Table S7 | Genes in the neighborhood of SNPs detected by the three methods (MRMLM, RMLM and EMMA) along with the results provided in the Arabidopsis information resource at http://www.arabidopsis.org/ and in Atwell *et al*. (2010)

**Trait**	**Gene detected**	**Chr**	**SNP (bp)**	**MRMLM**			**RMLM**			**EMMA in this study**			**EMMA in Atwell** ***et al*.(2010)**		**Arabidopsis Information Resource**	**Reference**
				**LOD**	**Effect**	**r^2 (%)**	**P-value**	**Effect**	**r^2 (%)**	**P-value**	**Effect**	**r^2 (%)**	**P-value**	**Effect**		
LD	*AT1G23000*	1	8128350	4.12	-0.055	0.56							1.25E-3	-0.143	√	
	SVP	2	9588685				4.47E-10	-0.397	23.36	2.78E-9	-0.407	24.59	2.78E-9	-0.407	√	Hartmann *et al*., 2000
	AGL17	2	9611587	8.83	-0.104	1.67	7.11E-7	-0.262	10.56				1.14E-6	-0.273	√	Han *et al*., 2008
	ETC3	4	454542	4.70	-0.066	0.72							3.17E-6	-0.223		Tominaga *et al*., 2008
	GA1	4	1260796	3.06	-0.059	0.63							2.30E-5	-0.210		Reeves *et al*., 2001
	AT4G14385	4	8291057	6.18	-0.060	0.66							5.11E-3	-0.118	√	
	FLC	5	3188328	9.48	-0.097	1.35	5.08E-7	-0.248	8.73	8.82E-7	-0.258	9.45	8.82E-7	-0.258		Hepworth *et al*., 2002
LDV	*CENH3*	1	164375	6.30	-0.049	3.37							4.07E-3	-0.052	√	
	SVP	2	9588685	10.30	-0.049	2.98	1.61E-6	-0.111	15.43				1.32E-6	-0.117	√	Hartmann *et al*., 2000
	CKB4	2	18446546	5.75	-0.053	2.05	2.66E-8	-0.152	16.80	5.74E-8	-0.158	18.15	5.74E-8	-0.158	√	Portolés & Más, 2007
	DOG1	5	18599929	7.25	-0.058	4.77	2.04E-7	-0.083	9.82	2.71E-7	-0.087	10.87	2.71E-7	-0.087		
SD	*AT1G52930*	1	19713470	6.21	-0.049	1.27							1.82E-3	-0.088	√	
	HEN2	2	2916675	3.83	0.030	0.53							5.73E-6	0.115	√	
	EDA8	4	153402	6.47	-0.042	1.13	2.46E-8	-0.132	11.26	6.74E-8	-0.136	12.00	6.74E-8	-0.136	√	

	ETC3	4	458226	10.70	-0.070	2.03	1.20E-6	-0.132	7.18				2.09E-6	0.115		Tominaga *et al*., 2008
	IDL3	5	3051259	6.23	-0.037	0.87							2.52E-4	-0.087	√	
	AT5G19430	5	6546055	4.66	-0.068	1.36							1.75E-3	-0.135	√	
W0	*AGL18*	3	21239134	6.24	-0.111	3.13							1.67E-3	-0.154	√	Gu *et al*., 2013
	DOG1	5	18592535				1.83E-6	-0.274	12.72				3.93E-6	-0.287		
	DOG1	5	18595015	11.87	-0.145	3.56							3.93E-6	-0.287		
W2	*ANP1*	1	2899659	8.11	-0.081	1.55							5.29E-3	-0.117	√	
	ETC3	4	454542	7.18	-0.089	1.72	4.30E-7	-0.207	9.21				8.07E-7	-0.215		Tominaga *et al*., 2008
	DCL4	5	6846957	3.36	-0.082	0.99							5.95E-4	-0.177	√	
W4	*ATPERK12*	1	8341601	4.65	-0.070	1.34							3.04E-4	-0.151	√	
	SVP	2	9588685				3.55E-9	-0.354	28.25	1.97E-8	-0.365	30.03	1.97E-8	-0.365	√	Hartmann *et al*., 2000
	AT2G30600	2	13031229	9.65	-0.107	3.00							1.12E-3	-0.163	√	
	C3HC4	5	6546259	5.94	-0.132	2.75							2.96E-5	-0.268	√	
	AT5G45190	5	18264316	7.06	-0.071	1.33							2.08E-4	-0.135	√	
	DOG1	5	18607728	10.09	-0.164	4.69	8.13E-7	-0.263	12.09				1.61E-6	-0.275		

LD: days to flowering under long days; LDV: days to flowering under long days with vernalization; SD: days to flowering under short days; W0: days to flowering under long days for no vernalization; W2: days to flowering under long days for 2 weeks vernalization; W4: days to flowering under long days for 4 weeks vernalization.

References

1 Han P, García-Ponce B, Fonseca-Salazar G, Alvarez-Buylla ER, Yu H. AGAMOUS-LIKE 17, a novel flowering promoter, acts in a FT-independent photoperiod pathway. Plant J. 55(2): 253-265 (2008)

2 Reeves, P., and Coupland, G. Analysis of flowering time control in Arabidopsis by comparison of double and triple mutants. Plant Physiol 126:1085-1091 (2001).

3 Portolés S, Más P. Altered oscillator function affects clock resonance and is responsible for the reduced day-length sensitivity of CKB4 overexpressing plants. Plant J. 51(6):966-77 (2007).

4 Hepworth, S., Valverde, F., Ravenscroft, D., Mouradov, A., and Coupland, G. Antagonistic regulation of flowering-time gene SOC1 by CONSTANS and FLC via separate promoter motifs. EMBO J 21:4327-4337 (2002).

5 Gu X, Wang Y, He Y. Photoperiodic regulation of flowering time through periodic histone deacetylation of the florigen gene FT. PLoS Biol. 11(9): e1001649 (2013).

6 Hartmann U, Höhmann S, Nettesheim K, Wisman E, Saedler H, Huijser P. Molecular cloning of SVP: A negative regulator of the floral transition in Arabidopsis. Plant J 21(4):351–360 (2000).

7 Tominaga R, Iwata M, Sano R, Inoue K, Okada K, et al. Arabidopsis CAPRICE-LIKE MYB 3 (CPL3) controls endoreduplication and flowering development in addition to trichome and root hair formation. Development 135: 1335–1345 (2008).

Table S8 | Comparison of various critical values (0.001, 0.01 and 0.05) using the multi-locus random-SNP-effect mixed linear model method (MRMLM)

QTL	True value				0.001			0.01			0.05		
	Chr.	Position (bp)	Effect	r^2 (%)	Power (%)	Effect	MSE	Power (%)	Effect	MSE	Power (%)	Effect	MSE
1	1	11298364	1.4762	10	95.0	1.5795	0.1143	94.8	1.5412	0.0701	94.6	1.5669	0.1342
2	1	11655607	1.0438	5	64.1	1.2136	0.0660	63.9	1.2083	0.0674	57.2	1.2357	0.0794
3	2	5066968	1.0438	5	38.9	1.3222	0.1343	49.3	1.3291	0.1423	40.3	1.3274	0.1550
4	2	5134228	1.8080	15	97.3	1.7994	0.1856	97.3	1.7922	0.0867	95.7	1.8203	0.2092
5	2	5464675	1.0438	5	46.6	1.3503	0.1501	42.7	1.3178	0.1318	38.0	1.3910	0.1831
6	2	6137189	1.0438	5	66.4	1.2428	0.0798	63.5	1.2072	0.0659	63.4	1.2323	0.0801
	False positive rate (0.01%)				0.75			1.68			2.51		

Table S9 | Comparison of powers and mean squared errors (MSE) for three GWAS methods (MRMLM, RMLM and EMMA) with the simulated genotypic data derived from the minPtest R package from the five simulation experiment where six QTNs were simulated.

QTN	True value			MRMLM			RMLM			EMMA		
	Position	Effect (SD)	r^2 (%)	Power (%)	Effect	MSE	Power (%)	Effect	MSE	Power (%)	Effect	MSE
1	763	2.5642(0.0972)	15	99.0	2.3340	0.2266	94.5	2.5286	0.1866	85.0	2.7033	0.1598
2	1455	2.0850(0.0846)	10	91.5	2.0365	0.1451	74.5	2.2459	0.1618	54.5	2.4718	0.2284
3	4402	1.4858(0.0629)	5	44.0	1.5904	0.0919	15.5	1.9309	0.2973	6.5	2.2609	0.6682
4	5500	1.4753(0.0630)	5	46.5	1.6133	0.1016	19.0	1.9403	0.2763	5.5	2.2316	0.5951
5	7410	1.4733(0.0567)	5	48.0	1.6339	0.1000	19.5	1.9445	0.2848	9.0	2.2168	0.5909
6	8507	1.4778(0.0663)	5	45.5	1.6352	0.1000	16.0	1.9757	0.3137	5.5	2.3230	0.7324

The fifth Monte Carlo simulation experiment

In the fifth simulation experiments, all the SNP genotypes were derived from the minPtest R package (Hieke et al. 2014). The sample size was 200 and the number of SNP markers was 10000. Six QTNs were simulated and placed on the SNP positions 763 (with the heritability of 0.15), 1455 (0.10), 4402

(0.05), 5500 (0.05), 7410 (0.05) and 8507 (0.05). The average was set at 10.0; and residual variance was set at 10.0. Although minor allele frequency was set at 0.30, three genotypes (AA, Aa and aa) were simulated so that the frequencies for three genotypes were calculated sample by sample. Thus, each QTN effect was calculated for each sample and standard deviation for true QTL effect was listed also in Table S9. The other was the same as those in the first simulation experiment.

Reference

Hieke S, Binder H, Nieters A, Schumacher M. minPtest: a resampling based gene region-level testing procedure for genetic case-control studies. *Computational Statistics* **29**(1-2), 51–63 (2014).

Software S1 | The program code for the multi-locus random-SNP-effect mixed linear model (MRMLM)

This file for the program code of the MRMLM includes the following files:

1) "input files”:

nk-1.xls is the K matrix;

nx-1.xlsx is genotypic values of each SNP marker for all the individuals. This file is too large, we cannot upload as *.xls file. If the reviewers and readers need this file, please contact with corresponding author Yuan-Ming Zhang (soyzhang@mail.hzau.edu.cn);

yy-1.xls is the phenotypic values for all the individuals; for example,

```
3.734689
3.258097
5.298317
4.419343
5.298317
3.373028
5.298317
5.298317
5.298317
5.298317
3.579073
3.516714
3.811281
  3.2711
3.314186
5.298317
5.298317
 3.65138
4.527584
3.465736
3.355736
3.847337
3.569727
3.308106
3.289644
3.455266
5.298317
4.944168
3.718472
```

3.238678
5.298317
5.298317
3.54578
5.060574
5.090764
3.314186
3.225522
3.361531
3.455266
4.050771
3.651198
3.807589
3.574607
3.433987
3.289644
3.829395
3.872069
3.620258
3.283416
3.289644
3.218876
3.455266
3.332205
3.693902
3.491656
3.785137
3.935902
3.792521
3.301992
3.638133
3.205454
3.840349
3.506767
3.912857
4.108275
5.298317
3.754068
3.289644
5.298317
5.298317
3.766033

4.267948
3.560296
3.387774
5.298317
3.433987
3.99713
3.344038
3.164069
3.149882
4.595456
3.142716
3.361531
4.090867
3.326233
3.60678
4.169889
3.807356
3.390025
3.385095
4.446972
3.501546
3.734357
3.924775
5.298317
5.298317
5.222192
4.623678
3.606592
3.593117
4.150121
4.156407
3.850739
3.449988
3.952045
3.218876
3.629145
3.516509
5.298317
5.298317
4.554316
3.726195
3.044522

3.574411
5.298317
3.349904
5.298317
5.298317
4.533048
3.533687
5.298317
5.298317
3.521652
3.361531
3.526361
3.620258
4.007938
3.218876
5.298317
3.520461
3.33814
4.143796
3.540959
5.298317
4.344347
3.579073
3.308106
3.378962
5.298317
3.566946
4.277245
3.258097
3.417727
3.395625
3.258097
3.380995
3.390025
3.734524
3.283416
3.709295
5.298317
4.407228
3.516509
5.298317
3.295837

3.536521
3.961607
3.314186
4.554316
3.460513
5.298317
3.733494
3.569826
3.491443
3.506557
3.301992
3.788756

2) "program code":

"step-1-R" is R code for singly-locus random-SNP-effect mixed linear model method.

Note: the input dataset files must be the *.csv format. The R codes are as follows.

```
dir<-"I:\\realdata"
setwd(dir)

rm(list=ls())
gen<-read.csv(file="nx-1.csv",header=FALSE)
phe<-read.csv(file="yy-1.csv",header=FALSE)
kk<-read.csv(file="nk-1.csv",header=FALSE)

mixed<-function(x,y,kk){

   loglike<-function(theta){
       lambda<-exp(theta)
       logdt<-sum(log(lambda*delta+1))
       h<-1/(lambda*delta+1)
       yy<-sum(yu*h*yu)
       yx<-matrix(0,q,1)
       xx<-matrix(0,q,q)
       for(i in 1:q){
          yx[i]<-sum(yu*h*xu[,i])
          for(j in 1:q){
             xx[i,j]<-sum(xu[,i]*h*xu[,j])
          }
       }
```

```
        loglike<- -0.5*logdt-0.5*(n-q)*log(yy-t(yx)%*%solve(xx)%*%yx)-0.5*log(det(xx))
        return(-loglike)
}

fixed<-function(lambda){
        h<-1/(lambda*delta+1)
        yy<-sum(yu*h*yu)
        yx<-matrix(0,q,1)
        xx<-matrix(0,q,q)
        for(i in 1:q){
              yx[i]<-sum(yu*h*xu[,i])
              for(j in 1:q){
                    xx[i,j]<-sum(xu[,i]*h*xu[,j])
              }
        }
        beta<-solve(xx,yx)
        sigma2<-(yy-t(yx)%*%solve(xx)%*%yx)/(n-q)
        sigma2<-drop(sigma2)
        var<-diag(solve(xx)*sigma2)
        stderr<-sqrt(var)
        return(c(beta,stderr,sigma2))
  }

  qq<-eigen(kk)
  delta<-qq[[1]]
  uu<-qq[[2]]
  q<-ncol(x)
  n<-ncol(kk)
  vp<-var(y)
  yu<-t(uu)%*%y
  xu<-t(uu)%*%x
  theta<-0
  parm<-optim(par=theta,fn=loglike,hessian = TRUE,method="L-BFGS-B",lower=-50,upper=10)
  lambda<-exp(parm$par)
  conv<-parm$convergence
  fn1<-parm$value
  fn0<-loglike(-Inf)
  lrt<-2*(fn0-fn1)
  hess<-parm$hessian
  parmfix<-fixed(lambda)
  beta<-parmfix[1:q]
  stderr<-parmfix[(q+1):(2*q)]
```

```
    sigma2<-parmfix[2*q+1]
    lod<-lrt/4.61
    p_value<-1-pchisq(lrt,1)
    sigma2g<-lambda*sigma2
    goodness<-(vp-sigma2)/vp
    par<-data.frame(lrt,beta,stderr,sigma2,lambda,sigma2g,lod,p_value)
    return(par)
}


loglike<-function(theta){
   xi<-exp(theta)
   tmp0<-zz*xi+1
   tmp<-xi*solve(tmp0)
   yHy<-yy-t(zy)%*%tmp%*%zy
   yHx<-yx-zx%*%tmp%*%zy
   xHx<-xx-zx%*%tmp%*%t(zx)
   logdt2<-log(det(tmp0))
   loglike<- -0.5*logdt2-0.5*(n-s)*log(yHy-t(yHx)%*%solve(xHx)%*%yHx)-0.5*log(det(xHx))
   return(-loglike)
}

fixed<-function(xi){
   tmp0<-zz*xi+diag(1)
   tmp<-xi*solve(tmp0)
   yHy<-yy-t(zy)%*%tmp%*%zy
   yHx<-yx-zx%*%tmp%*%zy
   xHx<-xx-zx%*%tmp%*%t(zx)
   zHy<-zy-zz%*%tmp%*%zy
   zHx<-zx-zx%*%tmp%*%zz
   zHz<-zz-zz%*%tmp%*%zz
   beta<-solve(xHx,yHx)
   tmp2<-solve(xHx)
   sigma2<-(yHy-t(yHx)%*%tmp2%*%yHx)/(n-s)
   gamma<-xi*zHy-xi*t(zHx)%*%tmp2%*%yHx
   var<-abs((xi*diag(1)-xi*zHz*xi)*as.numeric(sigma2))
   stderr<-sqrt(diag(var))
   result<-list(gamma,stderr,beta,sigma2)
   return(result)
}
name<-gen[,1:2]
gen<-gen[,3:(ncol(gen))]
```

```
gen<-t(gen)
n<-nrow(gen)
m<-ncol(gen)
x<-matrix(1,n,1)
ll<-numeric()
s<-ncol(x)
kk<-as.matrix(kk)
qq<-eigen(kk)
delta<-qq[[1]]
uu<-qq[[2]]
xu<-t(uu)%*%x
for(ii in 1:1)
{yy<-phe[,1]
  y<-as.matrix(yy)
  parm<-mixed(x=x,y=y,kk=kk)
  lambda<-parm$lambda[1]
  h<-1/(delta*lambda+1)
  yu<-t(uu)%*%y
  xx<-matrix(0,s,s)
  for(i in 1:s){
        for(j in 1:s){
              xx[i,j]<-sum(xu[,i]*h*xu[,j])
        }
  }
  yy<-sum(yu*h*yu)
  yx<-matrix(0,s,1)
  for(i in 1:s){
        yx[i]<-sum(yu*h*xu[,i])
  }

  qq<-numeric()
  for(k in 1:m){
        z<-as.matrix(gen[,k])
        zu<-t(uu)%*%z
        zy<-as.matrix(sum(yu*h*zu))
        zz<-as.matrix(sum(zu*h*zu))
        zx<-matrix(0,s,1)
        for(i in 1:s){
              zx[i]<-sum(xu[,i]*h*zu)
        }
        theta<-c(0)
        par<-optim(par=theta,fn=loglike,hessian = TRUE,method="L-BFGS-B",lower=-10,upper=10)
```

```
        xi<-exp(par$par)
        conv<-par$convergence
        fn1<-par$value
        hess<-par$hessian
        parmfix<-fixed(xi)
        gamma<-parmfix[[1]]
        stderr<-parmfix[[2]]
        beta<-parmfix[[3]]
        sigma2<-parmfix[[4]]
        lambda<-xi
        sigma2g<-lambda*sigma2
        fn0<-loglike(-Inf)
        lrt<-2*(fn0-fn1)
        p_lrt<-1-pchisq(lrt,1)
        wald<-(gamma/stderr)^2
        p_wald<-1-pchisq(wald,1)
        parm0<-c(ii,name[k,1],name[k,2],beta,sigma2,sigma2g,gamma,stderr,wald,p_wald)
        qq<-rbind(qq,parm0)
  }
  ll<-rbind(ll,qq)
}


parms<-ll
colnames(parms)<-c("trait","chr","pos","beta","sigma2","sigma2g","SNP_est","SNP_err","wald","p_wald")

write.table(x=parms,file="R1.csv",sep=",",row.names=FALSE)
```

The result file for R codes is "R1.csv", which is the result for the single-locus random-SNP-effect mixed linear model method. This result will be used in the SAS program.

In the "R1" file, the first column "trait" is the trait code, the second column "chr" is the chromosome code for the SNP marker, the third column "pos" is the SNP position (bp) on chromosome, the 4th column "beta" is the estimate for fixed effect β; the 5th column "sigma2" is the estimate for residual variance σ^2; the 6th column "sigma2g" is the variance of SNP effect (ϕ_k^2 in the manuscript), the 7th column "SNP_est" is the estimate for SNP effect ($\mathrm{E}(\gamma_k \mid \mathbf{y}^*)$); the 8th column "SNP_err" is the posterior variance of SNP effect ($\mathrm{var}(\gamma_k \mid \mathbf{y}^*)$) ; and the 9th and 10th columns, "wald" and "p-wald", are the Wald statistic and its P-value.

"step-2-SAS" is SAS code for the second step of multi-locus random-SNP-effect mixed linear model method.

Note: the input dataset files must be the *.csv format. The SAS codes are as follows.

```
%let dir=I:\realdata;
options ps=10200;
filename aa "&dir\nx-1.csv";
filename b1 "&dir\R1.csv";
filename cc "&dir\yy-1.csv";
filename dd "&dir\result1.csv";
data one;
    infile aa dlm=','   lrecl=1000000;
    input x1-x169;
run;
data c1;
    infile b1 dlm=','   lrecl=1000000 firstobs=2;
    input x1-x10;
run;
data three;
    infile cc dlm=','   lrecl=1000000;
    input x1-x1;
run;
proc iml symsize=90000000;


start ebayes_EM(x,z,y,b,u,v0,wang);
    n=nrow(z); k=ncol(z);
    if min(abs(eigval(x`*x)))<1e-6 then b=(x`*x+1e-8*i(ncol(x)))**-1*(x`*y);
    else b=(x`*x)**-1*(x`*y);
    v0=(y-x*b)`*(y-x*b)/n;
    u=j(k,1,0); v=j(k,1,0); s=j(k,1,0);

    do i=1 to k;
        zz=z[,i];
        s[i]=(zz`*zz)**-1*v0;             * The variance of QTL effect var(gamma);
        u[i]=s[i]*zz`*(y-x*b)/v0;         * The expectation of QTL effect E(gamma);
        v[i]=u[i]**2+s[i];                * The prior variance of QTL effect sigma(i);
    end;

    vv=j(n,n,0);do i=1 to k;zz=z[,i];vv=vv+zz*zz`*v[i];end;vv=vv+i(n)*v0;

    iter=0; err=1000; iter_max=100; err_max=1e-8;
    lambda=1;   * lambda is the lasso parameter;
```

```
        tau=0;            * tau is the prior belief of the scaled inverse chi-square;
        omega=0;      * omega is the scale of the inverse chi-square;

        do while (iter<iter_max & err>err_max);
            iter = iter+1;
            v01=v0;v1=v;b1=b;
            if min(abs(eigval(vv)))<1e-6 then vi=(vv+1e-8*i(ncol(vv)))**-1;
            else vi=vv**-1;
            if min(abs(eigval(x`*vi*x)))<1e-6 then b=inv(x`*vi*x+1e-8*i(ncol(x)))*(x`*vi*y);
            else b=inv(x`*vi*x)*(x`*vi*y);
            r=y-x*b;

            ss=j(n,1,0);
            do i=1 to k;
                zz=z[,i];
                u[i]=v[i]*zz`*vi*r;
                s[i]=v[i]*(1-zz`*vi*zz*v[i]);
                *v[i]= (sqrt(1+4*lambda*(u[i]**2+s[i]))-1)/(2*lambda);          * lasso(lamda);
                v[i]=(u[i]**2+s[i]+omega)/(tau+2+1);                              * scaled inverse chi-square;
                ss=ss + zz*u[i];
            end;
            v0=r`*(r-ss)/n;

            vv=j(n,n,0);do i=1 to k;zz=z[,i];vv=vv+zz*zz`*v[i];end;vv=vv+i(n)*v0;

            err=( (b1-b)`*(b1-b)+(v01-v0)**2+(v1-(v))`*(v1-(v)) )/(1+1+k);
            beta=b`;
            sigma2=v0;

        end;

        free wang;
        do i=1 to k;
            stderr=sqrt(s[i]+ 1e-20);
            t=abs(u[i])/stderr;
            f=t**2;
            p=1-probchi(f,1);
            wang=wang//(i||t||p);
        end;

    finish ebayes_EM;
```

```
    start lod0(xx,y,lod);
        n_sam=nrow(xx);
        nxb=ncol(xx);
        ww1=1:ncol(xx);
        lod=j(1,nxb,.);
        if ncol(ww1)>1.5 then do;
            ad=xx[,ww1];
            if abs(det(ad`*ad))<1e-6 then bb=inv(ad`*ad+i(ncol(ad))*0.01)*ad`*y;
            else bb=inv(ad`*ad)*ad`*y;
            vv1=(y-ad*bb)`*(y-ad*bb)/n_sam;
            ll1=sum(log(abs(pdf('normal',y,ad*bb,sqrt(vv1)))));

            sub=1:ncol(ww1);
            do ii=1 to ncol(ww1);
                ij=setdif(sub,ii);ad1=ad[,ij];
                if abs(det(ad1`*ad1))<1e-6 then bb1=inv(ad1`*ad1+i(ncol(ad1))*0.01)*ad1`*y;
                else bb1=inv(ad1`*ad1)*ad1`*y;
                vv0=(y-ad1*bb1)`*(y-ad1*bb1)/n_sam;
                ll0=sum(log(abs(pdf('normal',y,ad1*bb1,sqrt(vv0)))));
                lod[ww1[ii]]=-2.0*(ll0-ll1)/(2.0*log(10));
        end;end;
        do ii=2 to nxb; if lod[ii]=0 then do; lod[ii]=0; end;end;
    finish lod0;
use one; read all into x0;
use c1; read all into code;
use three; read all into yy;
cccc=x0[,1:2];
x0=x0[,3:ncol(x0)];

      hh=ncol(loc(code[,10]<=0.01));
      aa=(1:nrow(code))`||code[,10];
      call sort(aa,2);
      cc=aa[1:hh,];name=aa[1:hh,1];
      w0=code[,6]||code[,8];
      ww0=1-w0[,2]#w0[,2]/w0[,1];
      k0=loc(ww0<0);
      if ncol(k0)>0 then ww0[k0,1]=j(ncol(k0),1,0);
      nn=sum(ww0);
      pp=0.05/nn;
      a0=ncol(loc(aa[,2]<=pp));
      gg=name;
```

```
do ii=1 to nrow(name)-1;
do jj=ii+1 to nrow(name);
    if cccc[name[ii],1]=cccc[name[jj],1] then do;
        if abs(cccc[name[ii],2]-cccc[name[jj],2])<=20000 then gg[jj,1]=0;
    end;
end;
end;
free misfit kk kk0 l0;
if a0>0 then do;
    g0=gg[1:a0,1];
    kk0=a0;
    a0=loc(g0>0);
    g0=g0[a0,1];
    xxx0=(x0[g0,])`;
    call lod0(j(nrow(xxx0),1,1)||xxx0,yy,lod);
    lod=lod[2:ncol(lod)];
    kk=loc(lod>=1.5);
    kk1=loc(lod<1.5);
    if ncol(kk1)>0 then do;
        misfit=g0[kk1,1];
    end;
    if ncol(kk)>0 then do;
        g0=g0[kk,1];
        xx0=xxx0[,kk];
        lo=lod[kk,1];
    end;
    if ncol(kk)=0 then do;
        kk=0;
    end;
end;
else do;kk0=0;kk=0;end;
gg=gg[kk0+1:nrow(gg),1]//misfit;
a1=loc(gg>0);
a2=gg[a1,1];
xx=(x0[a2,])`;
if kk^=0 then xin=j(nrow(xx),1,1)||xx0;
else xin=j(nrow(xx),1,1);
call ebayes_EM(xin,xx,yy,b,u,v0,wang);
w2=loc(wang[,3]<=0.01);
free ww;
if ncol(w2)>0 then do;
    name=a2[w2,1];
```

```
          x3=xin||xx[,w2];
          call lod0(x3,yy,lod);
          lod=lod[2:ncol(lod)];
          w3=loc(lod>=3);
          if kk^=0 then name=g0//name;
          if ncol(w3)>0 then do;
               lo=lod[w3,1];
               ww=name[w3,];
          end;
          else ww=0;
     end;
     else do;
          yang=loc(lo>=3);
          if ncol(yang)>0 then do;ww=g0[yang,1];lo=lo[yang,1];end;
          else ww=0;
     end;

     if ww^=0 then do;
          ex=j(nrow(xx),1,1)||(x0[ww,])`;
          if abs(det(ex`*ex))<1e-6 then bbbb=inv(ex`*ex+i(ncol(ex))*0.01)*ex`*yy;
          else bbbb=inv(ex`*ex)*ex`*yy;
          eeff=bbbb[2:nrow(bbbb),1];
          wan=cccc[ww,]||eeff||lo;
     end;

create t1 from wan; append from wan;close t1;
quit;
proc export data=t1 outfile=dd dbms=csv replace;
run;
```

In the "result1" file from SAS codes, the first column "COL1" is the chromosome code, the second column "COL2" is the position (bp) of each SNP marker on chromosome, the third column "COL3" is the effect of SNP, and the last one "COL4" is the LOD score in the likelihood ratio test for the SNP marker.

The results are as follows.

COL1	COL2	COL3	COL4
4	429928	-0.07362	5.556129
5	3188328	-0.09746	9.484828
2	9611587	-0.10405	8.828852
5	25407568	-0.0746	5.196812
5	2047037	0.118348	9.115736

4	15645842	-0.16972	17.91585
4	454542	-0.06601	4.697095
4	1260796	-0.05925	3.06379
3	15980134	-0.04793	3.534653
5	19469740	-0.07981	6.115792
3	10857602	0.069876	5.602716
4	6265724	-0.05453	4.425055
4	8949791	0.066513	3.741759
4	17324466	0.056529	3.993891
3	7548675	0.055235	4.970269
1	16590690	0.071829	4.826888
2	3908570	-0.08401	8.382317
1	8128350	-0.05538	4.120952
5	21844046	-0.07226	8.821553
1	2779077	-0.09919	9.073548
1	19306376	-0.09155	8.925572
5	12782295	-0.05176	3.191567
4	15531205	0.071562	7.002315
4	2579595	-0.07774	5.286083
4	8291057	-0.06023	6.179478
1	11738770	0.05638	4.605569
4	6904769	-0.07102	6.645415
4	109403	-0.07116	5.369578
5	23928370	-0.09638	9.975715
